# An End-to-End Pipeline for Early Diagnosis of Acute Promyelocytic Leukemia Based on a Compact CNN Model

**DOI:** 10.3390/diagnostics11071237

**Published:** 2021-07-11

**Authors:** Yifan Qiao, Yi Zhang, Nian Liu, Pu Chen, Yan Liu

**Affiliations:** 1 The College of Computer Science, Sichuan University, Chengdu 610065, China; qiaoyifan@stu.scu.edu.cn (Y.Q.); yzhang@scu.edu.cn (Y.Z.); 2 The College of Electrical Engineering, Sichuan University, Chengdu 610065, China; liu-nian@scu.edu.cn; 3 The Department of Laboratory Medicine, Zhongshan Hospital, Fudan University, Shanghai 200032, China

**Keywords:** acute promyelocytic leukemia, convolutional neural networks, early diagnosis, pipeline, real cases validation

## Abstract

Timely microscopy screening of peripheral blood smears is essential for the diagnosis of acute promyelocytic leukemia (APL) due to the occurrence of early death (ED) before or during the initial therapy. Screening manually is time-consuming and tedious, and may lead to missed diagnosis or misdiagnosis because of subjective bias. To address these problems, we develop a three-step pipeline to help in the early diagnosis of APL from peripheral blood smears. The entire pipeline consists of leukocytes focusing, cell classification and diagnostic opinions. As the key component of the pipeline, a compact classification model based on attention embedded convolutional neural network blocks is proposed to distinguish promyelocytes from normal leukocytes. The compact classification model is validated on both the combination of two public datasets, APL-Cytomorphology_LMU and APL-Cytomorphology_JHH, as well as the clinical dataset, to yield a precision of 96.53% and 99.20%, respectively. The results indicate that our model outperforms the other evaluated popular classification models owing to its better accuracy and smaller size. Furthermore, the entire pipeline is validated on realistic patient data. The proposed method promises to act as an assistant tool for APL diagnosis.

## 1. Introduction

Acute promyelocytic leukemia (APL) is one of the sub types of acute myeloid leukemia (AML), namely M3, according to the French–American–British (FAB) classification guidelines of acute leukemia. The incidence of APL is around 0.23 per 100,000 people in China and around 600–800 cases each year in the United States [1]. It accounts for 10% to 15% of the present AML cases [2]. The susceptible population of APL are adults, with more than 30% of patients being over 60 years old, but recently the number of reported pediatric patients is rising [3,4].

Early diagnosis plays a dramatically important role in APL treatment selection and is closely bound to its prognosis, since APL benefits most from timely treatment compared with the other subtypes of AML. APL used to be fatal before the specific target therapy with all-trans retinoic acid (ATRA) was adopted. Currently the five-year disease free survival rate has risen from 25% to 95%, and APL has been thought to be almost a curable disease. Despite this, it is still difficult to avoid early death (ED) before or during the initial therapy due to mortal hemorrhagic complications such as disseminated intravascular coagulation (DIC) [5]. Meanwhile, the occurrence of ED increases with age [6]. Theoretically speaking, the diagnosis of APL should consist of microscopy examination of peripheral blood and bone marrow cells, flow cytometry analysis and advanced genetic or molecular level confirmation. However, in order to avoid the fatal hemorrhagic complications, morphologically suspected APL patients should be hospitalized and treated with proper therapy as soon as possible regardless of whether the molecular confirmation is obtained or not [7]. Thus, the initial treatment plan is usually generated only based on screening through the microscopy examination of peripheral blood smears. It is actually a critical step in the entire diagnosis–treatment flow and is expected to be sensitive and fast.

According to the morphology screening guidelines for APL proposed by FAB, the microscopic examination should be implemented manually by well-trained hematopathologists, which are scarce in most primary hospitals [8]. Besides, objective bias is difficult to avoid in manual work, which may lead to missed diagnosis and misdiagnosis. It may affect treatment plans, resulting in a completely different clinical outcome. According to the recommendations of the International Council for Standardization in Haematology (ICSH), enough nucleated cells should be analyzed for each peripheral blood sample to obtain a meaningful clinical deterministic conclusion [9]. The target cell needs to be sought first through a low power microscopic lens, then their appearance should be analyzed at high magnification. Furthermore, APL often causes very low white blood cell counts in the early stage of the disease; it requires staff to spend a lot of time identifying rare promyelocytes from the whole smear through the microscope. Therefore, the manual check is a tedious and cumbersome job and the bottleneck of the entire diagnosis–treatment workflow. Current guidelines encourage people to pay attention to fast and convenient morphological methods for the early diagnosis of APL [10]. It would be of great clinical significance if there was a sensitive and automatic system to help analyze peripheral blood smears for APL. However, as far as we know, there is currently little research focusing on the automatic identification and diagnosis of APL. That is what inspired us to solve this problem via artificial intelligence.

There have been state-of-the-art achievements in the classification or automatic detection of cell images of acute leukemia, especially for acute lymphoblastic leukemia (ALL) and AML. These methods categorize all the cells and then predict the leukemia based on the existence of abnormal blast cells, some of which segment the individual white blood cells first. The main differences among them are the different classification algorithms employed. Some of them chose the traditional solutions to classification problems, for example, workflow consisting of manually-designed image features and support vector machines as the classifier [11,12,13] or using hybrid hierarchical classifiers [14] or the Adaboost algorithm with random forest as a classifier [15]. Due to the important role of the extracted features in the final classification, some studies [16,17,18] investigated different methods to select the most appropriate features. Besides, the detection step combined with classification is proposed which can directly work on the whole blood smears [19,20]. However, the performance of these methods depends largely on the quality of the extracted features. The other methods utilized the advanced convolutional neural networks (CNN) with transfer learning techniques or the generative adversarial optimization algorithm to learn image features automatically and to classify the cells simultaneously in one pipeline [21,22,23]. A predictive model with two cascaded CNNs was designed as an assistant tool to help the clinical pathologists in the diagnosis of acute leukaemia during the process of blood smear review [24]. These methods may need fine tuning from benchmark datasets due to the lack of cell images.

In addition, there are some cell image processing, analysis and segmentation methods based on deep learning proposed for problems other than acute leukemia. By comparing the prediction of two classifiers and selecting the label with the higher confidence value, the overall accuracy of embryo image classification has been further increased [25]. A deep learning method based on Region Based CNN and a central coordinate tracking algorithm were proposed for the detection of sperm head [26]. For the cell images taken directly from cell culture flasks with a benchtop microscope, an unsupervised clustering method—Self-Label Clustering—was introduced to identify different morphological phenotypes within a cell type [27].

In this study, we develop a clinically applicable pipeline to help with the early diagnosis of APL. According to the FAB guidelines, the most critical evidence of APL is the discovery of abnormal promyelocytes in peripheral blood through a series of morphological features including kidney-shaped or biloba nuclei, large and/or numerous cytoplasmic granules, and Auer rods [28]. A compact network architecture based on the attention embedded CNN blocks is designed to accurately distinguish promyelocytes from normal cells and to generate an initial opinion for diagnosis. Our contributions are listed as follows:

1. A new compact cell classification model based on the attention embedded convolutional neural network is designed to distinguish promyelocytes from the normal white blood cells. The whole model is tiny and it is easily trained on a small dataset to avoid fine tuning. The experiment’s results demonstrate that it outperforms other popular benchmark classification models.
2. To the best of our knowledge, this is the first work to investigate the early diagnosis method of APL on a large dataset consisting of all public accessible datasets and private data. The entire dataset is nearly twice as large as that used in current work and includes multicenter data, which helps with generalization.
3. The end-to-end pipeline has been validated on realistic patient data.

The remainder of the paper is organized as follows: Section 2 explains the details of the dataset, the proposed methodology of the pipeline, the architecture of our model and the metrics. The results of the classification model, entire workflow, case report and ablation study are presented in Section 3. Section 4 discusses the merits and limitations of our method and Section 5 concludes the study.

## 2. Materials and Methods

**Figure 1:**
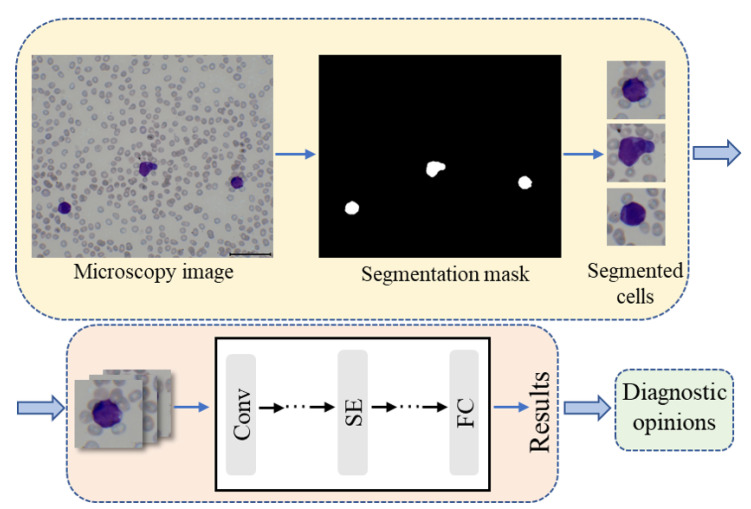
The flowchart of the proposed artificial intelligence aided workflow for APL diagnosis, which includes leukocyte focusing, cell classification and diagnostic opinions steps.

The proposed artificial intelligence aided pipeline for APL diagnosis works on the microscopy images of peripheral blood smear samples. It consists of three steps, namely leukocyte focusing, cell classification and diagnostic opinions as shown in Figure 1. First, the target cells are extracted from the microscopic images via a color feature based segmentation method. Second, the individual cell images are fed into the proposed classification model to identify cell types. Finally, the diagnostic opinions are given according to the classification results obtained by step 2 and the clinical prior knowledge.

### 2.1. Leukocytes Focusing

The input of our system is the microscopy image, which contains numerous blood cells. Since we mainly care about leukocytes in APL diagnosis, the first step is to focus on our target cells. As shown in Figure 1, the appearance of leukocytes is quite different from that of other cells, as they are large and conspicuous during the preparation of smears. Hereby, the color feature based cell extraction method proposed in our prior work was employed [29]. The microscopy images were first operated by color normalization to align their means and standard deviations to the selected reference images, which aimed to reduce the interference caused by staining. Then color deconvolution was utilized to deconvolve the normalized image into the methylene blue stain space and the eosin stain space. This follows the assumption that the compound staining image is the linear combination of the monochrome stain. Finally, the deconvolved methylene blue stain images were binarized to generate the bounding box of the leukocytes and localize the cells. The details of the cell extraction method can be found in the reference [29].

### 2.2. Cell Classification

**Figure 2:**
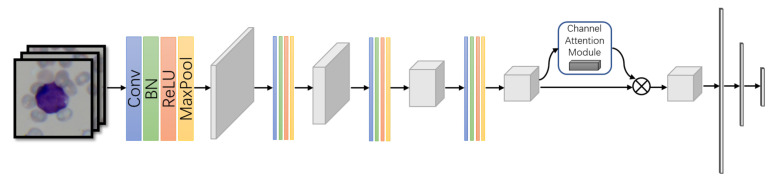
The architecture of the proposed compact model, containing four convolution blocks, one channel attention module and two fully-connected layers.

A compact CNN model with attention mechanism embedding was designed to perform the cell classification. It consists of a group of convolution layers—a channel attention module and two fully-connected layers as shown in Figure 2.

A convolution layer is composed of convolution, batch normalization, ReLU activation function and max pooling operation as listed in Equation (1).

(1) $$F\left(\cdot \right)={Pooling}_{Max}\left(ReLU\left(BN\left(Conv\left(\cdot \right)\right)\right)\right),$$

where F(·) denotes the feature maps generated by the convolution layer and Conv(·) describes the convolution operation. BN(·), ReLU(·) and ${Pooling}_{Max}\left(\cdot \right)$ are the batch normalization, ReLU function and max pooling, respectively. The small convolution kernel of 3×3 was applied to balance the performance and the computational cost. The dropout layer with dropout rate set to 0.5 was added here after each fully-connected layer to prevent the over-fitting problem.

Since the convolution group produced a large number of feature maps, the channel-wise attention module was embedded to highlight the most discriminative feature channels and help them to contribute more to the final prediction. It was implemented through the squeeze-and-excitation (SE) module [30] as in Equation (2).

(2) $$\left\{\begin{array}{c} F_{sq}\left(\cdot \right)=Pooling_{channel-wise}\left(F\left(\cdot \right)\right);\\ F_{ex}\left(\cdot \right)=SoftMax\left(W_{2}ReLU\left(W_{1}F_{sq}\right)\right), \end{array}\right.$$

where $F_{sq}\left(\cdot \right)$ performs the channel-wise global average pooling on the feature map F(·) generated by the convolution group. $F_{ex}\left(\cdot \right)$ is the generated weights for each channel. $W_{1}$ and $W_{2}$ are the matrices that first map the $F_{sq}\left(\cdot \right)$ into a low dimension space and then recover it back. The compression ratio aims to balance the attention capacity and computational cost of the SE block and was set to 16.

Two cascaded fully-connected layers were employed to compress the feature maps and to generate the final prediction for cell classification. The details of the architecture are shown in Table 1.

### 2.3. Diagnostic Opinions

Based on the latest National Comprehensive Cancer Network (NCCN) guidelines, the induction therapy should be started as soon as possible in cases suspected of APL by morphological examination [31]. Thus, we sought to produce a diagnosis opinion to warn of a suspicious status if there was a positive result in our classification step. It can be provided at the same time as the risk stratification since it is one of the prognosis factors and a guidance for consolidation therapy. The risk was calculated with the Sanz/PETHEMA and GIMEMA-risk score [28].

### 2.4. Dataset

Three datasets were employed to validate the performance of the proposed model. The first was the public dataset AML-Cytomorphology_LMU, which contains more than 10,000 expert-labeled single-cell images taken from peripheral blood smears diagnosis with acute myeloid leukemia at Munich University Hospital [32]. The second was the public dataset from [33], which includes peripheral blood smear images of 106 patients at The Johns Hopkins Hospital (JHH). Taking into consideration that both datasets contain patients from several subtypes of AML, the typical APL corresponding to six kinds of cells were chosen to construct the subset in our paper, named APL-Cytomorphology_LMU and APL-Cytomorphology_JHH and including 14,910 and 7695 leukocytes, respectively. These two datasets were mainly used to validate the cell classification model since they were already single-cell images and did not need a focusing step. Additionally, 6798 leukocytes—as the clinical dataset for validating the whole pipeline—were extracted according to the method in [29] from 657 microscopy images. These images were collected from the blood smears in the hematology lab of Zhongshan Hospital, Fudan University. Some were diagnosed with APL and the others were from normal individuals because of the rare appearance of the other five kinds of normal cells in the blood smears of APL patients. All images were obtained from the peripheral blood smears by microscope and were manually labeled by experienced hematologists following the standard clinical protocol. The entire cell image distribution of the three datasets is listed in Table 2. We randomly divided images in each dataset into five folds. Stratified split was carried out so that each fold contained approximately 20% of the cell images of each class and had no duplicate images with the other folds. Each model was trained from scratch for five rounds individually. In each round, three folds were the training set, one fold was the validation set and the remaining one fold was taken as the testing set. Note that the testing set was different in each of the five rounds. The result was acquired by averaging the testing results of the five rounds. Random data augmentation included flip and rotation applied to the training set to relieve the data imbalance problem. Finally, three case reports were provided to show the end-to-end performance in terms of the early diagnosis of APL. The data used in the case reports did not overlap with those in any aforementioned dataset.

### 2.5. Metrics

In order to measure the performance of our method quantitatively, the most commonly used statistics, denoted as the true positives TP, the false positives FP, the true negatives TN and the false negatives FN, were employed. Based on them, we calculated the metrics listed in Table 3. ROC analysis was performed through *TPR* and *FPR*.

### 2.6. Experiment Setup

The entire system was implemented through Python and the cell classification framework was based on Pytorch [34]. All experiments were conducted on a work station equipped with one NVIDIA GTX 1080Ti GPU and an AMD Ryzen 5 1600 processor.

In the training phase of the cell classification model, the hyper parameters were as follows. The SGD optimizer was applied with momentum = 0.9. The initial learning rate was set as $5\times 10^{-3}$ and was divided by 5 every 10 epochs. The drop out method was applied in our network to prevent the overfitting problem with a drop rate equal to 0.5. Instead of fine tuning, all the models were trained from scratch.

## 3. Results

### 3.1. Performance of Cell Classification Model

**Figure 3:**
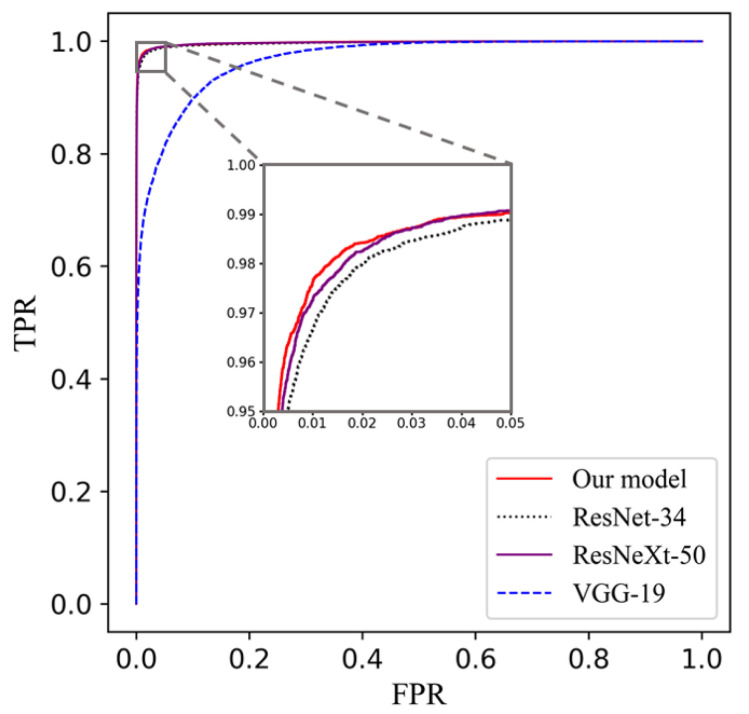
ROC curves of six kinds of cell classification results of the four models on the APL-Cytomorphology_LMU dataset; our model achieved an AUC of 0.9977 ± 0.0003, obtained by averaging the testing results of five different models trained for cross-validation. Each testing set accounts for approximately 20% of the dataset and does not have repetitive cell images.

In order to validate the performance of our proposed compact cell classification model, we trained our model on the APL-Cytomorphology_LMU and the APL-Cytomorphology_JHH. The results are listed in Table 4 and Table 5. Our model was compared with ResNet [35], ResNeXt [36] and VGG net [37], which are famous classification models that achieved superior performances on blood cell classification tasks. For the sake of fairness, the architecture of all models followed their original papers and were trained on the same data partitioning. Based on Table 4 and Table 5, the area under the curve (AUC) of each model is very close except for VGG-19; our model yields the leading performance in all remaining metrics while having a relatively small number of parameters. It can be read from Table 6 that the size of our model is around one third of that of ResNet-34 or ResNeXt-50, and less than one tenth of VGG-19. Consequently, it has lower demands on the size of the training dataset and takes less time to train. The receiver operating characteristic (ROC) curve for each model is plotted in Figure 3 and the confusion matrices are given in Table 7 and Table 8.

**Figure 4:**
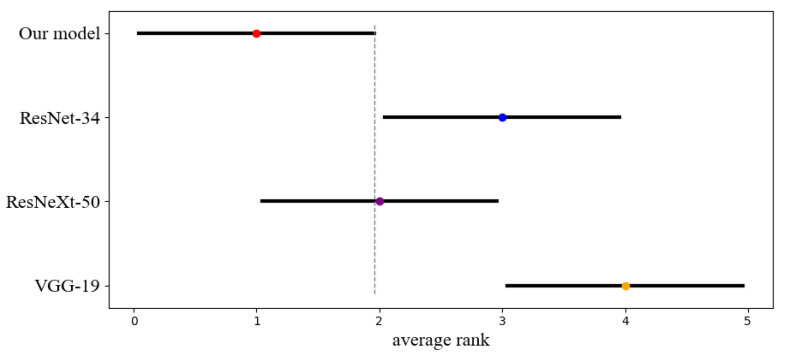
The difference between the average ranks of four models using the Critical Distance diagrams made from the results of the post-hoc Nemenyi test (α=0.05). The dot shows its average rank value, and the horizontal line with the dot at the center shows the size of its CD (1.915).

We performed the statistical analysis of the classification results using the non-parametric Friedman test and obtained a *p*-value = 0.0018, which is smaller than 0.05, to prove that the performance differences of the four models are statistically significant. We then visualized the difference between the average ranks of the four models using the Critical Distance (CD) diagrams made from the results of the post-hoc Nemenyi test (α=0.05) as shown in Figure 4. The dot shows its average rank value, and the horizontal line with the dot at the center shows the size of its Critical Distance. Our model is significantly better than the ResNet-34 and VGG-19.

Based on the ROC curves, the VGG-19 performed poorly compared to the other three models, and there was a little improvement of our model on the APL-Cytomorphology_LMU dataset, achieving an AUC of 0.9977, compared with those of ResNet-34 and ResNeXt-50. Clinically, some leukemia cells of variant APL are easily confused with monocytes due to their sparse granules and distorted nuclei. Similar results could be found from the confusion matrix in Table 7 and Table 8, that some promyelocytes were misclassified as monocytes.

**Figure 5:**
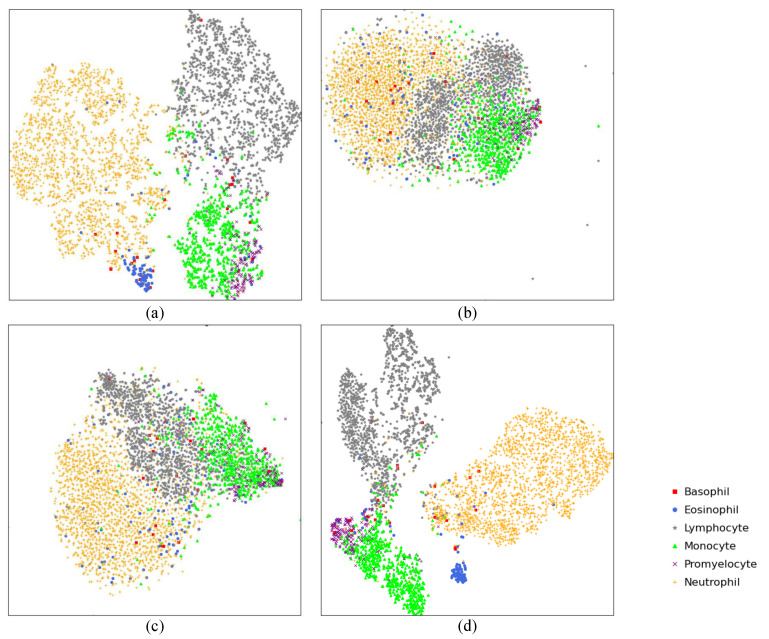
The learned feature of the four models visualized through t-SNE. For each image in the testing set, we save the parameters of the last fully-connected layer during the inferencing process. The dimensions of the last fully-connected layer of our network and ResNet-34 are 512; the ResNeXt-50’s is 2048 and the VGG-19’s is 4096. The dimension of the embedded space was set to 2 for better visualization and PCA was applied to initialize the embedding space. (**a**) Our model; (**b**) ResNet-34; (**c**) ResNeXt-50. (**d**) VGG-19.

The learned features of the four networks were visualized to show the learning ability of different networks on our task through the t-SNE method [38]. The high dimensional features obtained by the last layer of each model were projected to the Cartesian coordinates as plotted in Figure 5. The features extracted by ResNet-34 and ResNeXt-50 did not achieve gratifying divisibility due to the overlaps. However, for VGG-19, several eosinophil and basophil scatters located inside the neutrophil cluster that causes the misclassification. It revealed the reason why the classification performance of VGG-19 is relatively poor.

### 3.2. Performance of the Entire Workflow

We validated the entire workflow designed for the early diagnosis of APL on the clinical dataset, from the cell focusing step to the diagnosis step. Before performing the end-to-end study, we first trained the cell classification model on the clinical dataset following the same experiment settings listed in the results section. The classification performance is listed in Table 9 and the confusion matrix is shown in Table 10.

### 3.3. Case Report

Three patient cases were presented here to validate the performance of our early diagnosis system. The patients were divided into three categories based on their Sanz/PETHEMA and GIMEMA-risk score. The definition of risk stratification is given in Table 11.

The first patient was a 42-year-old female who was diagnosed as APL with low risk. The peripheral blood smear was acquired on the same day of diagnosis. The WBC was equal to $2.1\times 10^{9}/$L and platelet count was around $63\times 10^{9}/$L, calculated by the hematologist. Based on our pipeline, 149 leukocytes were extracted automatically through 153 visual fields of the smear. The second patient was a 54-year-old male who was diagnosed as APL with intermediate risk. The peripheral blood smear was acquired the day before diagnosis. The WBC was equal to $7.3\times 10^{9}/$L and the platelet count was around $27\times 10^{9}/$L, calculated by the hematologist. Based on our pipeline, we automatically extracted 198 leukocytes through 70 visual fields of the smear. The third patient was a 77-year-old female who was diagnosed as APL with high risk. The peripheral blood smear was acquired the day before diagnosis. The WBC was equal to $25.5\times 10^{9}/$L and the platelet count was around $16\times 10^{9}/$L, calculated by the hematologist. Based on our pipeline, 98 leukocytes were extracted automatically through 15 visual fields of smears. The cell distribution of the three patients is listed in Table 12.

The end-to-end performance of the entire workflow is given in Table 13. Based on the results of the classification model, the yielded sensitivity and specificity are clinically acceptable at our institution. The diagnosis opinion is also given in the form of a potential treatment plan according to the risk and diagnosis results obtained by our workflow. The potential treatment for patient 1 and patient 2 is ATRA and arsenic; patient 3 needed chemotherapy induction besides ATRA and arsenic, which is consistent with the real clinical treatment. To be more specific, the promyelocytes account for a large proportion of the data of patient 3, and there are only eight other leukocytes in total. For the class of promyelocyte, the TNR is low while the proportion is high, therefore the final TNR becomes very low when the weighted sum method is applied to calculate the metrics.

### 3.4. Ablation Study of the Classification Model

In this section, we investigate the effectiveness of dropout layer, SE layer and the influence of the number of convolution blocks on the final classification performance of the proposed model on the combination of two public datasets. The model-NoSE means the proposed model without the SE layer; model-NoDropout is the proposed model without the dropout layer; model-2ConvBlock and model-3ConvBlock are models with different convolution blocks.

As shown in Table 14, we can observe that the SE layer and the dropout layer can improve the performance for about 0.0063 and 0.0026 of $F_{\beta }$score, respectively, and the learning ability of the model is not strong enough if fewer convolution blocks are applied.

## 4. Discussion

In this paper, we demonstrated an end-to-end pipeline for the early diagnosis of APL based on a compact CNN model. APL used to be considered one of the most dangerous acute leukemias with shockingly high early mortality due to bleeding complications. Recently, with the development of the molecular biological pathogenesis of the disease and the breakthrough of all-trans retinoic acid (ATRA) combined with arsenic trioxide, the cure rate of APL has been greatly improved. Therefore, early screening, early diagnosis and timely treatment are particularly important in clinical practice. In the 2019 NCCN clinical guidelines, it recommends that the retinoic acid induction therapy should be applied as soon as possible once APL is suspected based solely on the morphology characteristics, since early clinical intervention is important for decreasing the early bleeding mortality and improving the long-term cure rate. As an important means of initial diagnosis, the morphological analysis of biological smears through microscopy has high technical requirements for hematologists, and faces problems such as long training cycle, poor repeatability, strong subjectivity and high rate of missed detection, especially in primary hospitals. What is worse is that most APL cases have a low white blood cell count, which makes the manual check more difficult. We seek to provide a potential solution to address the mentioned difficulties. There are three components, including the leukocyte, that focus on identifying the white blood cells from the microscopic images: the cell classification to classify the abnormal promyelocytes and the five types of normal leukocytes, as well as the final diagnosis recommendation associated with the risk estimation.

The popular classification networks require more computing resources during the training process due to the large number of parameters of these networks. For our task, not only are there the problems of slow training and inferencing, but also the risk of overfitting. Instead of using these networks, a compact convolutional neural network model embedded with the channel-wise attention mechanism was proposed. The model has fewer tunable parameters so that it does not ask for fine-tuning based on the large public dataset and has lower hardware requirements. However, the achieved performance is competitive as shown in Section 3. For the sake of fairness, a comparison with the popular classification models is performed on a subset of the public dataset AML-Cytomorphology_LMU and the dataset APL-Cytomorphology_JHH, which include all the APL corresponding cells. As shown in Table 4 and Table 5, the proposed cell classification model achieves the leading performance for most metrics. Additionally, the classification performance of abnormal promyelocytes is validated in our clinical dataset. From the confusion matrix shown in Table 10, we can see that there is only one misclassification from abnormal promyelocyte to monocyte. The experiment’s results, validated on multicenter data, show that our model is sensitive and robust enough for the most critical cells.

Since the two public datasets only provide microscopic images of single cells, we chose to validate our entire workflow on the clinical data and to investigate its capability of auxiliary early diagnosis. This time, we trained the cell classification model for use in the workflow on the clinical dataset from scratch to avoid any domain bias. The classification performance is listed in Table 9. From the confusion matrix, the model misclassifies 11 cells while still keeping high accuracy and high sensitivity for the abnormal promyelocytes. Additionally, case reports are provided to show the end-to-end diagnosis suggestion. The peripheral blood smear samples from three APL patients with different risks are accessed. The data are not included in the clinical dataset used to train the model. The microscopic images of the mentioned smears are the input of our workflow. The leukocyte focusing method crops each individual white blood cell to feed the classification model. Based on the cell classification model, the sensitivity of the abnormal promyelocyte reached 99.21%, and the precision for the total of the other five types of normal cells also reached more than 99%. The final diagnosis is given, associated with the risk. Due to the different situations of the patients, we have to acquire a different number of visual fields to obtain enough leukocytes for diagnosis. As the distribution of leukocytes changes, the visual fields we need are adjusted. For example, for patient 1, there are only around one to two leukocytes in a blood smear image due to the clinical symptoms of leukocyte decline caused by APL, and more than 100 view fields have to be reviewed to yield a decision. For patient 3, 15 visual fields are enough. Due to this, the processing time varies for each patient because a different number of view field images have to be loaded. The time for cell classification is almost stable for each patient because the number of target leukocytes required for diagnosis is similar.

**Figure 6:**
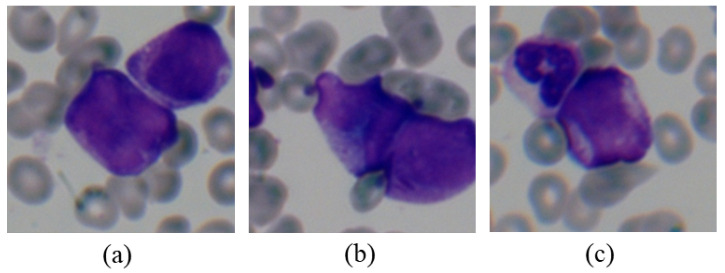
Three overlapping cell samples extracted from patient 3. The four cells in (**a**,**b**) are all promyelocytes, the upper one in (**c**) is neutrophil, and the other is the promyelocyte.

The limitation of the proposed pipeline is that the overlap of cells occurs frequently in the peripheral blood smears of high-white-blood patients, due to the dense distribution of white blood cells, as shown in Figure 6. This leads to the possibility of dividing two or more cells into one field of view and results in misclassification, which might be solved by further investigating the cell segmentation method.

## 5. Conclusions

In this study, we demonstrated an artificial intelligence aided APL diagnosis pipeline consisting of cell focusing, classification and diagnostic opinions. The cell focusing step helps to find the leukocytes from the microscopic images through automatic segmentation methods. The presented compact convolutional neural network model embedded with an attention mechanism can identify the abnormal promyelocytes directly from microscopy images through cell classification. The model distinguishes the cells based on the automatic learned features, instead of on the manually designed ones. The experiment’s results demonstrate that our model achieves a better performance than the competitive models, such as ResNet-34 and ResNeXt-50, on both public datasets—APL-Cytomorphology_LMU and APL-Cytomorphology_JHH—as well as on the clinical dataset. Our method can be employed in situations with very low white blood cell counts at the early stage of APL to save time when making a diagnosis decision. As an entire workflow, the proposed method shows great potential to act as a tool for the early diagnosis of APL.

## Figures and Tables

**Table 1:** The detailed structure of our classification model.

Layer	Operation	Filter	Stride	Padding	Output Size
Block0	Conv	3 × 3 × 64	1	1	112×112×64
	BN, ReLU	-	-	-	
	Max pool	3×3	2	1	
Block1	Conv	3×3×128	1	1	56×56×128
	BN, ReLU	-	-	-	
	Max pool	3×3	2	1	
Block2	Conv	3×3×256	2	1	14×14×256
	BN, ReLU	-	-	-	
	Max pool	3×3	2	1	
Block3	Conv	3×3×256	1	1	7×7×256
	BN, ReLU	-	-	-	
	Max pool	3×3	2	1	
SE	-	-	-	-	7×7×256
FC0	-	-	-	-	512
FC1	-	-	-	-	6

**Table 2:** The amount of six classes of leukocytes of the three datasets.

Dataset	Basophil	Eosinophil	Lymphocyte	Monocyte	Promyelocyte	Neutrophil
APL-Cytomorphology_LMU	79	424	3937	1789	88	8593
APL-Cytomorphology_JHH	53	108	3435	1319	688	2092
Clinical dataset	953	1063	976	912	628	2282

**Table 3:** The quantitative metrics.

Metric	Definition
Sensitivity	$TPR=\frac{TP}{TP+FN}$
Specificity	$TNR=\frac{TN}{TN+FP}$
False positive rate	$FPR=\frac{FP}{TN+FP}$
False negative rate	$FNR=\frac{FN}{TP+FN}$
Precision	$Precision=\frac{TP}{TP+FP}$
$F_{\beta }$score	$F_{\beta }$score $=\left(1+\beta^{2}\right)\frac{Precision\cdot TPR}{(\beta^{2}\cdot Precision)+TPR}$

**Table 4:** The performance of the four models on the APL-Cytomorphology_LMU dataset by 5-fold cross-validation.

Method	TPR	TNR	FPR	FNR	Precision	$F_{\beta }$score	AUC
Our model	**0.9746**±**0.0048**	**0.9905 ± 0.0015**	**0.0095 ± 0.0015**	**0.0254 ± 0.0048**	**0.9765 ± 0.0035**	**0.9756 ± 0.0039**	**0.9977 ± 0.0003**
ResNet-34 [35]	0.9680 ± 0.0043	0.9874 ± 0.0020	0.0126 ± 0.0020	0.0320 ± 0.0043	0.9679 ± 0.0056	0.9680 ± 0.0045	0.9970 ± 0.0005
ResNeXt-50 [36]	0.9719 ± 0.0034	0.9891 ± 0.0018	0.0109 ± 0.0018	0.0281 ± 0.0034	0.9690 ± 0.0032	0.9705 ± 0.0031	0.9975 ± 0.0006
VGG-19 [37]	0.9425 ± 0.0144	0.9292 ± 0.0078	0.0208 ± 0.0078	0.0575 ± 0.0144	0.9438 ± 0.0168	0.9431 ± 0.0154	0.9698 ± 0.0140

**Table 5:** The performance of the four models on the combination of the two public datasets by 5-fold cross-validation.

Method	TPR	TNR	FPR	FNR	Precision	$F_{\beta }$score	AUC
Our model	**0.9658 ± 0.0031**	**0.9881 ± 0.0011**	**0.0120 ± 0.0011**	**0.0342 ± 0.0031**	**0.9653 ± 0.0030**	**0.9655 ± 0.0031**	0.9914 ± 0.0026
ResNet-34 [35]	0.9557 ± 0.0048	0.9837 ± 0.0020	0.0163 ± 0.0020	0.0443 ± 0.0048	0.9549 ± 0.0043	0.9553 ± 0.0045	0.9921 ± 0.0008
ResNeXt-50 [36]	0.9594 ± 0.0016	0.9854 ± 0.0025	0.0146 ± 0.0025	0.0406 ± 0.0016	0.9589 ± 0.0017	0.9591 ± 0.0016	**0.9944 ± 0.0006**
VGG-19 [37]	0.9363 ± 0.0068	0.9756 ± 0.0026	0.00304 ± 0.0145	0.0637 ± 0.0068	0.9347 ± 0.0070	0.9355 ± 0.0069	0.9674 ± 0.0060

**Table 6:** The number of parameters and floating point operations (FLOPs) of the four models.

Method	Params (M)	FLOPs (G)
Our model	**7.397**	**1.380**
ResNet-34 [35]	21.288	3.761
ResNeXt-50 [36]	22.992	4.257
VGG-19 [37]	139.595	19.634

**Table 7:** The confusion matrix of the classification performance of the four models for the testing data of the APL-Cytomorphology_LMU dataset acquired by rounding off the mean value of five results of 5-fold cross-validation to the nearest whole number (e.g., 0.4 is 0, 0.6 is 1); each number represents the amounts of cells classified.

Our Model/ResNet-34/		Predicted Class					
ResNeXt-50/VGG-19		Basophil	Eosinophil	Lymphocyte	Monocyte	Promyelocyte	Neutrophil
True class	Basophil	**9**/5/	0/1/	1/1/	1/1/	0/0/	4/7/
		6/3	1/3	1/2	1/1	0/0	6/6
	Eosinophil	1/1/	**79**/77/	1/0/	0/1/	0/0/	3/5/
		1/2	78/61	1/1	1/4	1/1	3/14
	Lymphocyte	0/0/	0/0/	**773**/768/	10/13/	0/0/	3/5/
		1/1	0/1	772/758	10/16	1/1	3/9
	Monocyte	0/0/	0/0/	9/8/	**340**/338/	1/3/	6/7/
		0/0	0/5	10/14	339/325	2/2	6/10
	Promyelocyte	0/0/	0/0/	0/0/	2/3/	14/14/	0/0/
		0/0	0/1	0/1	2/5	**15**/10	0/0
	Neutrophil	1/0/	1/1/	6/6/	5/9/	0/1/	**1705**/1701/
		1/2	1/8	8/10	8/8	1/1	1699/1689

**Table 8:** The confusion matrix of the classification performance of the four models for the testing data of the combination of two public datasets acquired by rounding off the mean value of five results of 5-fold cross-validation to the nearest whole number (e.g., 0.4 is 0, 0.6 is 1); each number represents the amounts of cells classified.

Our Model/ResNet-34/		Predicted Class					
ResNeXt-50/VGG-19		Basophil	Eosinophil	Lymphocyte	Monocyte	Promyelocyte	Neutrophil
True class	Basophil	**11**/8/	3/2/	3/3/	0/1/	3/4/	6/8/
		10/5	2/3	3/4	1/2	2/3	7/8
	Eosinophil	1/1/	**85**/81/	2/2/	2/2/	4/4/	11/15/
		2/3	83/64	2/3	2/6	2/3	15/27
	Lymphocyte	1/1/	1/0/	**1446**/1434/	18/24/	2/4/	6/10/
		1/4	2/1	1440/1417	20/30	4/2	7/20
	Monocyte	0/1/	1/0/	17/19/	**580**/569/	15/18/	9/14/
		1/2	0/4	17/28	572/545	19/25	12/16
	Promyelocyte	1/2/	1/0/	3/3/	19/27/	**131**/122/	1/0/
		2/2	1/1	2/4	22/29	127/118	1/1
	Neutrophil	2/2/	2/3/	9/12/	10/13/	2/3/	**2111**/2104/
		3/5	3/13	12/20	13/15	2/2	2103/2082

**Table 9:** The performance of the proposed method on the clinical dataset.

Method	TPR	TNR	FPR	FNR	Precision	$F_{\beta }$score
Our model	0.9919	0.9988	0.0012	0.0081	0.9920	0.9920

**Table 10:** The confusion matrix of the classification performance of the four models for the testing data of the combination of two public datasets; each number represents the number of cells classified.

		Predicted Class					
		Basophil	Eosinophil	Lymphocyte	Monocyte	Promyelocyte	Neutrophil
True class	Basophil	**190**	0	0	0	0	0
	Eosinophil	1	**210**	1	0	0	0
	Lymphocyte	0	0	**191**	2	2	0
	Monocyte	0	0	1	**180**	1	0
	Promyelocyte	0	0	0	1	**125**	0
	Neutrophil	0	0	0	2	0	**454**

**Table 11:** The APL risk stratification.

Risk	Metric	Patient ID
Low	$WBC\leq 10\times 10^{9}/$L, platelet count $>40\times 10^{9}/$L	1
Intermediate	$WBC\leq 10\times 10^{9}/$L, platelet count $\leq 40\times 10^{9}/$L	2
High	$WBC>10\times 10^{9}/$L	3

**Table 12:** The number of six types of leukocytes extracted from three patients.

Patient	Basophil	Eosinophil	Lymphocyte	Monocyte	Promyelocyte	Neutrophil
Patient 1	0	0	19	4	23	103
Patient 2	0	0	39	2	119	35
Patient 3	0	0	5	0	90	3

**Table 13:** The end-to-end diagnosis performance of three patients.

Patient	TPR	TNR	FNR	Precision	$F_{\beta }$score	Potential Treatment
Patient 1	0.8792	0.9548	0.1208	0.8732	0.8762	ATRA + arsenic
Patient 2	0.8974	0.9332	0.1026	0.8798	0.8885	ATRA + arsenic
Patient 3	0.9184	0.1964	0.0816	0.8521	0.8840	ATRA + arsenic + chemotherapy induction

**Table 14:** The performance of the ablation study on the combination of the two public datasets by 5-fold cross-validation.

Method	TPR	TNR	FPR	FNR	Precision	$F_{\beta }$score
Our model	**0.9658 ± 0.0031**	**0.9881 ± 0.0011**	**0.0120 ± 0.0011**	**0.0342 ± 0.0031**	**0.9653 ± 0.0030**	**0.9655 ± 0.0031**
model-NoSE	0.9607 ± 0.0042	0.9868 ± 0.0017	0.0132 ± 0.0017	0.0393 ± 0.0042	0.9577 ± 0.0058	0.9592 ± 0.0049
model-NoDropout	0.9633 ± 0.0032	0.9869 ± 0.0005	0.0131 ± 0.0005	0.0368 ± 0.0032	0.9627 ± 0.0033	0.9629 ± 0.0032
model-2ConvBlock	0.9539 ± 0.0049	0.9832 ± 0.0017	0.0168 ± 0.0017	0.0461 ± 0.0046	0.9490 ± 0.0051	0.9514 ± 0.0049
model-3ConvBlock	0.9606 ± 0.0040	0.9852 ± 0.0004	0.0148 ± 0.0004	0.0392 ± 0.0040	0.9600 ± 0.0040	0.9602 ± 0.0040

## Data Availability

The two public datasets are openly available under references [32,33]. The other part of the data presented in this study are available on request from the corresponding author. The data are not publicly available due to the ethical restrictions and privacy.
